# Floral morph variation mediated by clonal growth and pollinator functional groups of *Limonium otolepis* in a heterostylous fragmented population

**DOI:** 10.1093/aobpla/plae020

**Published:** 2024-03-27

**Authors:** Dengfu Ren, Fangfang Jiao, Aiqin Zhang, Jing Zhao, Jing Zhang

**Affiliations:** Xinjiang Key Laboratory of Biological Resources and Genetic Engineering, School of Life Science and Technology, Xinjiang University, Urumqi 830017, P.R China; Xinjiang Key Laboratory of Biological Resources and Genetic Engineering, School of Life Science and Technology, Xinjiang University, Urumqi 830017, P.R China; Xinjiang Key Laboratory of Biological Resources and Genetic Engineering, School of Life Science and Technology, Xinjiang University, Urumqi 830017, P.R China; Xinjiang Key Laboratory of Biological Resources and Genetic Engineering, School of Life Science and Technology, Xinjiang University, Urumqi 830017, P.R China; Xinjiang Key Laboratory of Biological Resources and Genetic Engineering, School of Life Science and Technology, Xinjiang University, Urumqi 830017, P.R China

**Keywords:** Clonal plant, floral morph variation, heterostyly, heteromorphic incompatibility, homostyly, *Limonium otolepis*, reciprocal herkogamy, sexual reproduction

## Abstract

**Abstract**. Heterostyly, a genetic style polymorphism, is linked to symmetric pollen transfer, vital for its maintenance. Clonal growth typically impacts sexual reproduction by influencing pollen transfer. However, the floral morph variation remains poorly understood under the combined effects of pollinators and clonal growth in heterostyly characterized by negative frequency-dependent selection and disassortative mating. We estimated morph ratios, ramets per genet and heterostylous syndrome and quantified legitimate pollen transfer via clonal growth, pollinators and reciprocal herkogamy between floral morphs in *Limonium otolepis*, a fragmented population composed of five subpopulations in the desert environment of northwestern China, with small flower and large floral morph variation. All subpopulations but one exhibited pollen-stigma morphology dimorphism. The compatibility between mating types with different pollen-stigma morphologies remained consistent regardless of reciprocal herkogamy. Biased ratios and ramets per genet of the two mating types with distinct pollen-stigma morphologies caused asymmetric pollen flow and varying fruit sets in all subpopulations. Short-tongued insects were the primary pollinators due to small flower sizes. However, pollen-feeding *Syrphidae* sp. triggered asymmetry in pollen flow between high and low sex organs, with short-styled morphs having lower stigma pollen depositions and greater variation. Clonal growth amplified this variation by reducing intermorph pollen transfer. All in all, pollinators and clonal growth jointly drive floral morph variation. H-morphs with the same stigma-anther position and self-incompatibility, which mitigate the disadvantages of sunken low sex organs with differing from the classical homostyly, might arise from long- and short-styled morphs through a ‘relaxed selection’. This study is the first to uncover the occurrence of the H-morph and its associated influencing factors in a distylous plant featuring clonal growth, small flowers and a fragmented population.

## Teaser

Floral morph variation was studied based on the investigation of pollen transfer mediated by clonal growth and pollinator functional groups in *Limonium otolepis* . It was shown that short-tongued insects were the primary pollinators due to small flower sizes. However, pollen-feeding Syrphidae sp. triggered asymmetry in pollen flow between high and low sex organs and promoted the occurrence of H-morph with self-incompatibility. It differed from the classical homostyly and might arise from long- and short-styled morphs through a ‘relaxed selection’. This study is the first to uncover the occurrence of the H-morph and its associated influencing factors in a distylous plant featuring clonal growth, small flowers and a fragmented population.

## Introduction

The fixed growth habits of plants increase the complexity and diversity of their reproductive processes. Compared to selective pressures on pollen dispersal, seed germination and seedling growth in sexual reproduction, clonal growth has clear advantages in terms of space and resource use and escape from environmental risks. Consequently, it is one of the most common modes of asexual reproduction in angiosperms (Klimeš *et al.* 1997). However, this leads us to probe the underlying mechanisms that facilitate the coexistence of the two distinct reproductive modes. This also raises the question of how natural forces select between them and what the ecological factors are governing the equilibrium between these modes. Whether clonal growth unilaterally affects sexual reproduction or whether the selective pressure arising from sexual reproduction simultaneously shapes clonal growth has been an important question in the field of the reproductive ecology of clonal plants (Zhang and Zhang 2006).

Heterostyly is a floral polymorphism in angiosperms featuring two (distyly) or three (tristyly) mating types differing reciprocally in stigma and anther height, in which there are short-styled morph (S-morph) and long-styled morph (L-morph) in distyly, and S-morph, L-morph and medium-styled morph (M-morph) in tristyly. Heterostylous plants have common traits, including moderate-sized flowers, reliance on long-tongued (LT) pollinators, radial corollas, long corolla tubes with concealed nectar at the base and low sexual organs within the tube (Gander 1979). LT insects are often ideal pollinators for maintaining isoplethic floral morph frequencies in populations, whereas short-tongued (ST) pollinators are typically ineffective (Arroyo and Dafni 1995; Arroyo *et al.* 2002; Furtado *et al.* 2023). For instance, the coastal loss of S-morph flowers in *Eichhornia azurea* (Pontederiaceae) may be linked to the absence of LT pollinators (Alves-dos-Santos 2002). Due to limited mating types and the strict pollinator selectivity associated with the long corolla tube, heterostyly is often variable or unstable (Barrett 2019; Zhong *et al.* 2019; Zhang *et al.* 2021), manifesting as floral morph deviation, loss of reciprocal herkogamy and breakdown of heteromorphic incompatibility systems, etc. (Wu *et al.* 2015; Zhou *et al.* 2015).

Heterostyly is considered an adaptation that promotes accurate disassortative pollen transfer through reciprocal herkogamy between morphs, realizing pollen deposition on the different parts of the pollinator’s body surface (Darwin 1877). Symmetrical disassortative pollen transfer between high and low sex organs is essential for the maintenance of heteromorphic incompatibility systems. Exuberant clonal propagation can impact sexual reproduction and plant life history. It may reduce male and female fitness through geitonogamy, blocking pollen flow, and causing sex ratio deviations due to clonal patches, and affecting resource allocation (Liu *et al.* 2009). In heterostylous plants, clonal growth’s effects on sexual reproduction are complex due to negative frequency-dependent selection and disassortative mating. For instance, in distylous *Nymphoides peltata* (Menyanthaceae), plants closer to compatible mating types within clonal patches show higher fruit sets, maintaining the heterostylous syndrome despite floral morph frequency deviations (Wang *et al.* 2005). Strong population substructure from clonal propagation may drive dioecism evolution from distyly in *Nymphoides* species (Ornduff 1966). In tristylous aquatic *Eichhornia azurea* (Pontederiaceae), clonality and weak self-incompatibility in mid-styled flowers make them more susceptible to geitonogamy due to clonal growth, leading to population isoplethy deviations (da Cunha *et al.* 2014). These studies reveal diverse effects of clonal growth on heterostylous plants, making it a crucial factor in studying heterostyly variation and evolution. It interferes with the function of heterostyly by compromising legitimate intermorph pollinations and competes with sexual reproduction.

However, previous research has primarily focussed on the impact of clonal growth on sexual reproduction (Eckert *et al.* 1999; Wang *et al.* 2005; Brys *et al.* 2007), genetic diversity and genetic structure of populations (Liao *et al.* 2013), floral morph frequency variation due to environmental heterogeneity (da Cunha *et al.* 2014), or geographical and demographic factors (Costa *et al.* 2017) and only in aquatic plants. Considering the difficulty of observing the visiting behaviour in aquatic plants, there has not been much attention on the influence of pollinators on pollen transfer and the joint effects with clonal growth on floral morph variation in natural populations (Eckert and Barrett 1993; Eckert *et al.* 1999; Eckert 2000; Brys *et al.* 2007). *Limonium otolepis* (Plumbaginaceae), a desert-dwelling perennial herb with clonality, features a kind of floral morph with matched pistil and stamen lengths (H-morph) that is widely distributed in a fragmented population based on a previous investigation, resembling self-compatible homostyly, excluding L-morph and S-morph floral morph. It provides model systems for investigating the influence of pollinators on pollen transfer and the joint effects with clonal growth on floral morph variation, as well as the relationship between clonal growth and sexual reproduction.

Our study aimed to address two main questions: (i) What are the clonal growth habits and heterostylous syndrome characteristics of *L. otolepis*? To answer this first question, we investigated the distribution of genets, average ramets per genet (clonal strength), floral composition, and the frequency of five subpopulations. Additionally, we examined aspects of the heterostylous syndrome, including floral size, ancillary polymorphism, and heteromorphic incompatibility. This phenomenon has also been reported by phylogenetic reconstructions based on 121 species in Plumbaginaceae (Costa *et al.*, 2019). (ii) How did the pollinator functional groups and clonal growth influence pollen transfer and floral morph variation? To answer this question, we first assessed the correlation between stigma-anther separation within a flower and pollen deposition on stigma based on a survey of pollinator species and pollination efficiency. We also analysed the impact of pollinators on floral morph variation. Second, we examined the effect of clonal growth on pollen transfer by comparing pollen deposition on stigma and fruit set within clonal patches consisting of a single floral morph and different floral morphs. These studies allowed us to explore the relationship between clonal growth and sexual reproduction, as well as the roles of pollinators and clonal growth in heterostyly variation and evolution.

## Materials and Methods

### Study species and site

**Figure 1.**
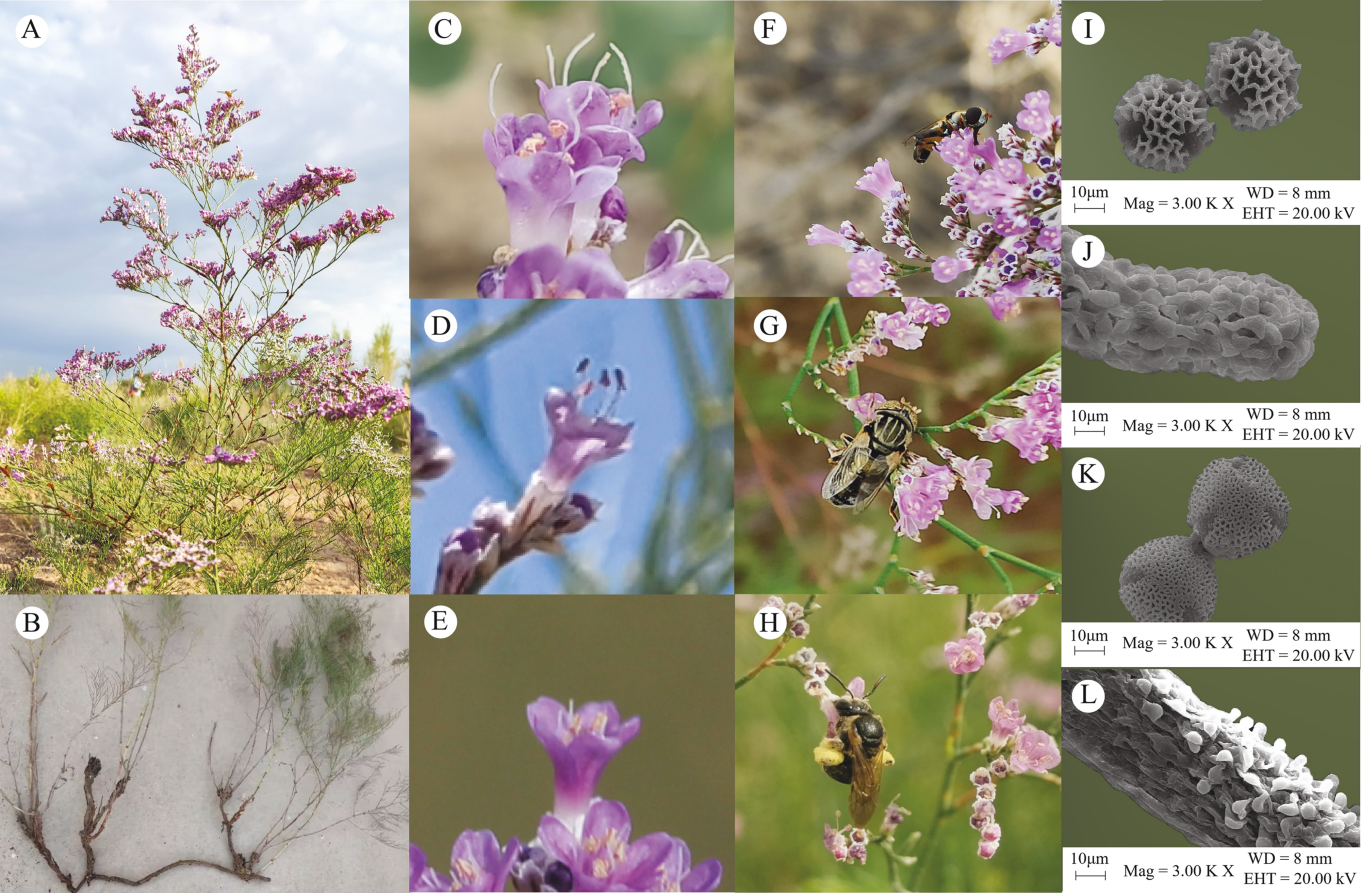
Clonal ramet, floral morphs, pollinators, and stigma-pollen morphology of *Limonium otolepis*. (A) flowering branch; (B) clonal ramet; (C), (D), (E) L-, S- and H-morph flower; (F), (G), (H) *Syrphidae* sp., *Muscidae* sp., and *Halictus* sp.; (I), (J) pollen grains and stigma morphology of L- and H_L_-morph (with cob-like stigma epidermal cells and coarse-reticulated pollen outer wall); (K), (L) pollen grains and stigma morphology of S- and H_S_-morph (with papillate stigma epidermal cells and fine-reticulated pollen outer wall).

*Limonium otolepis* (Plumbaginaceae) is a perennial, clonal, distylous plant, reaching heights of 30–90 cm (Fig. 1A and B). It produces 2–5 (7) loose spikelets on slender branches, each containing one or two purplish flowers with five pistils and five stamens. The filiform stigmas account for approximately 1/6 of the total pistil length in the L-morph and 1/3 that in the S-morph (Fig. 1C and D). Flowering occurs from June to July, with each ovary having one locule and one ovule. Fruits are enveloped by a persistent calyx and ripen from July to August.

**Figure 2.**
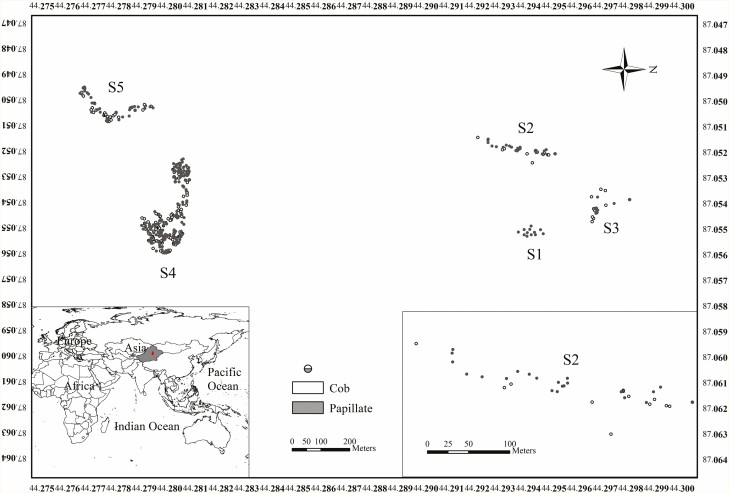
Distribution of different subpopulations and genets of *Limonium otolepis.*

This study was centred on a fragmented population comprising five subpopulations which comprised one subpopulation dominated by H-morphs and four mixed subpopulations with H-, L- and S-morphs. The population is located in a saline desert area within Hutubi County, Xinjiang, Northwest China (44°17ʹ13ʹʹ N, 87°3ʹ10ʹʹ E, H = 471 m) (Fig. 2), characterized by extensive clonal growth and discernible patchy clonal fragments. In mixed subpopulations with H-, L- and S-morph flowers, there are some plants with the coexistence of H- and S-morph flowers or H- and L-morph flowers, except for L-, H- and S- morph plants.

### Heterostylous syndrome and clonal growth

#### Floral morph composition and frequency across subpopulations

During the flowering season, we investigated all genet in the five subpopulations for floral morphology and floral morph frequency analysis by randomly selected ten flowers from each flowering genet. Floral morph frequency is expressed by the ratio of the number of certain floral morph to the total number of counted flowers in subpopulations. As there were two distinct pollen/stigma morphologies between L- and S-morph flowers, H-morphs with the same stigma morphology and pollen ornamentation as the L-morphs were named H_L_-morph flowers, whereas those with the same stigma epidermal cell morphology and pollen ornamentation as the S-morphs were named H_S_-morph flowers. Plants exclusively bearing H_L_-morph flowers were categorized as H_L_-morph individuals, while those with only H_S_-morph flowers were classified as H_S_-morph individuals. For H-morph identification, we used a microscope (Nikon, ECLIPSE E200, Nikon Corporation, Japan) to observe pollen or stigma morphology.

#### Distribution pattern and clonal strength of genet

During the flowering season of *L. otolepis*, we surveyed five subpopulations (S1–S5) to record genet/clonal fragment numbers, ramet counts per genet, and floral morph distributions. We also conducted genet localization to map their distribution and clonal growth patterns. Given the scattered and sporadic distribution of most genets, the consistent floral morphology and linear arrangement of ramets within the same genet, and the distinct pistil-stamen positions, pollen, and stigma morphologies, it was relatively straightforward to identify the genets. In clonal patches featuring H-morphs, we confirmed genets and their ramets by examining stigma morphology using a light microscope (Nikon, ECLIPSE E200, Nikon corporation, Japan).

#### Ancillary polymorphism and flower size parameters

To examine pollen and stigma morphology, we randomly chose 10 individuals per floral morph in each subpopulation, collecting 1–2 nearly open floral buds from each ramet. Anthers were air-dried in EP tubes, while stigmas were fixed in 2 % glutaraldehyde fixative (0.1 mol L^–1^ phosphate buffer). A scanning electron microscope (LEO 1430 VP, Carl Zeiss, Oberkochen, Germany) was used for studying pollen and stigma morphology.

For assessing size parameters and floral morph variation in the *L. otolepis* population, we randomly marked 15 × 4 individuals, each representing L-, S-, H_L_- and H_S_- morphs, from the S4 subpopulation. From each marked individual, 2–4 flowers were randomly sampled. We measured various floral characteristics, such as total flower length, corolla length, corolla tube length, corolla opening diameter, corolla tube diameter and stigma and anther heights. Because the five stamens within individual flowers often differed in length, we measured the tallest and shortest stamens in each flower and calculated the average anther height. A principal component analysis was then performed on these floral parameters.

To capture variations in stigma and anther heights, we selected 30 individuals from mixed clonal patches with two mating types exhibiting different pollen/stigma morphology (including L- and H_L_-morphs and S- and H_S_- morphs). From each of these individuals, 3–5 flowers were randomly chosen for measuring stigma and anther heights. Stigma and anther height distributions were subsequently analysed using a digital calliper with 0.02 mm accuracy.

#### Detection of heteromorphic incompatible systems

Owing to the occurrence of H-morph with different pollen/stigma morphology, there were four floral morphs (L-, S-, H_L_- and H_S_-morphs) in the population. To test the compatibility between floral morphs, 15 L-, S-, H_L_- and H_S_-morphs plants were labelled randomly. Before anther dehiscence, the pollen of various floral morphs were collected in the labelled plants and placed in different EP tubes for pollination treatments. Meanwhile, about 20 flowers were selected randomly from each plant for the following treatments (2–3 flowers per treatment): (i) apomixis (emasculated and netted); (ii) intramorph pollination and netted (L × L, S × S, H_L_ × H_L_, and H_S_ × H_S_); (iii) ‘intermorph pollination’ and netted (different from the traditional meaning of intermorph pollination) (L × S, S × L, L × H_L_, L × H_S_, S × H_S_, S × H_L_, H_S_ × L, H_S_ × S, H_S_ × H_L_, H_L_ × S, H_L_ × L, H_L_ × H_S_); (iv) artificial self-pollination and netted and (v) control (flowers were naturally pollinated). We assessed fruit sets in the various pollination treatments after fruit ripening. Additionally, we tested compatibility in a monomorphic S1 subpopulation of H-morph (H_S_-morph). We randomly labelled 15 individuals, each with 16 flowers, and subjected them to four treatments (four flowers per treatment): (i) intramorph pollination and netted, (ii) artificial self-pollination and netted, (iii) apomixis (emasculated and netted) and (iv) control (flowers were naturally pollinated). Subsequently, we counted the fruit set for each treatment upon ripening.

### Legitimate pollen transfer mediated by pollinator functional groups

#### Pollinator functional groups and visiting frequency

During the flowering period, 15 individuals of different floral morphs were labelled randomly in each subpopulation, with 3–7 plants selected randomly each day, and 1–2 flowering branches were labelled for each individual. From 8:30 to 14:00, we observed the pollinator species and visiting behaviour for half an hour per plant each time. The number of open flowers in the branch labelled and the number of flowers visited were recorded, and the flower-visiting frequency was calculated. The cumulative observation time of S1–S4 subpopulations was no less than 2 days.

#### Pollen deposition after single visit

In the S4 subpopulation, we investigated pollen transfer between floral morphs using mixed clonal patches. Given that each flower blooms for only a day, opening in the morning and closing in the afternoon, we took measures to control pollinator visits. We randomly selected 1–2 branches and netted them on selected individuals of different mating types in the afternoon to prevent insects from accessing the flowers ahead of time. The following morning, we opened the nets to allow pollinator visits. After a single visit, we removed the visited flowers to assess the stigma pollen deposition and discouraged the pollinator from visiting other flowers on the same plant. Additionally, we documented the insect species and the floral morphs of the visited flowers. We conducted these observations and samplings over 5 days. Because there are two distinct pollen-stigma morphologies in population, it is convenient to identify stigmatic illegitimate (incompatible) and legitimate (compatible) pollens of any floral morphs. For example, it is compatible between papillate stigma and coarse-reticulated pollen or between cob-like stigma and fine-reticulated pollen, while it is incompatible between papillate stigma and fine-reticulated pollen or cob-like stigma and coarse-reticulated pollen. To assess the stigma pollen deposition, after removing the visited flower, the style is gently stripped using tweezers and placed on a glass slide to make a temporary slide (Costa *et al.* 2017). We counted the number of legitimate and illegitimate pollen under a microscope based on the dimorphism of pollen-stigma morphology between mating types (Fig. 2).

#### Degree of herkogamy and pollen deposition

To study the relationship between stigma-anther separation and pollen deposition, we selected mixed clonal patches in the S4 subpopulation. From 15 representative plants per floral morph, we randomly sampled 2–3 flowers per individual and measured stigma and anther heights with a digital calliper. Subsequently, we calculated the absolute and average stigma-anther separation (ABS; AVG) using the parameters of the stigma height (S), the highest anther height (hA), the lowest anther height (lA), and the average anther height (aA), where ABS = S–hA, AVG = S–aA in flowers with cob-like stigma and ABS = lA–S, AVG = aA–S in flowers with papillate stigma. We assessed legitimate and illegitimate pollen grains on stigmas and analysed the correlation between stigma-anther separation and pollen grain numbers. The assessment of the stigma pollen deposition was as mentioned in ‘*Pollen deposition after single visit*’.

### Legitimate pollen transfer mediated by clonal growth

#### Impacts of the distance to the nearest opposite mating partner on pollen transfer

To assess the effect of clonal fragment size on pollen transfer, we chose a significant monomorphic patch (with papillate stigma epidermal cells) next to a patch featuring opposite mating genets (with cob-like stigma epidermal cells) in the S4 subpopulation. We sourced legitimate pollen (P0) from the nearest opposite mating genet with multiple ramets. We designated five sampling locations (P1–P5) within the patch, varying in distance from the source genet (0.5, 5.1, 14.3, 20.8 and 26.5 m), and marked 3–5 ramets at each location. After 5 h of pollen dispersal, we randomly selected 2–4 flowers from each marked ramet and quantified legitimate and illegitimate pollen grains on the stigma. The assessment of the stigma pollen deposition was as described in ‘*Pollen deposition after single visit*’. This sampling process was repeated daily for three consecutive days at each location.

#### Impacts of mating type ratio on pollen transfer and sexual reproduction

Mating type refers to floral morphs with different genotypes in a population, and generally there are two or three mating types in heterostylous population. For *L. otolepis*, there are two mating types: flowers with cob-like stigma (L- and H_L_-morph) and flowers with papillae stigma (S- and H_S_-morph). The mating types ratio is the proportion of flowers with cob-like stigma or papillae stigma in the population. To investigate the ecological impact of clonal growth induced mating type ratio deviations, we selected mixed and monomorphic patches of various sizes and mating type ratios. We recorded ramet numbers and pollen-stigma morphology in each patch. In each clonal patch, we randomly marked 15 ramets of each mating type, marking all ramets if they were scarce. After 5 h of flowering (pollen dispersal), we selected 2–5 flowers randomly from each marked ramet. We counted legitimate and illegitimate pollen grains on the stigma for a mating type ratio and stigma pollen deposition correlation analysis. The assessment of the stigma pollen deposition was as described in ‘*Pollen deposition after single visit*’. Additionally, we marked 3–5 branches on sampled ramets and counted fruit sets after ripening to correlate mating type ratios with fruit sets.

### Statistical analysis

We examined floral morph frequency and average ramets per genet using chi-square tests for deviations from isoplethy (1:1).

Floral traits of floral morphs were compared using generalized linear mixed-effects model (GLMMs) with a gaussian distribution using ‘identity’ link function, and using the ‘glmer’ function in the ‘lme4’ package in R (Bates *et al.* 2015). Morphs were used as predictive variables, floral traits as response variables, and different plants as random factors. The ‘check_normality’ function in the ‘performance’ package in R was used to check for normality (Lüdecke *et al.* 2021), If the residuals did not follow a normal distribution, GLMMs with a gaussian distribution using the ‘log’ link function was used to improve the normality of the residuals. The significance of each factor was tested with a type II analysis of deviance, which was conducted using the ‘Anova’ function in the ‘car’ package (Fox and Weisberg 2019). The ‘emmeans’ function with a Tukey’s adjustment in the package ‘emmeans’ (Lenth 2023) was used as a *post-hoc* test to detect significant differences between morphs. In addition, floral traits for different floral morphs were subjected to Principal Components Analysis (PCA) with R v4.2.3, along with the ‘factoextra’ (Kassambara and Mundt 2020) and ‘tidyverse’ packages (Wickham *et al.* 2019).

All fruit sets were analysed using GLMMs with a binomial distribution using the ‘logit’ function. To investigate the effects of treatment, morph, and their interaction on heteromorphic incompatibility, fruit set was modelled. Morph, treatment and their interactions were used as predictive variables, and different plants as random factors. To investigate the effects of mating type ratio in mixed patches on sexual reproduction, fruit set was modelled with stigma morphology and mating type ratio as a fixed factor, and ramet nested within different days as a random factor. The analyses were conducted using the ‘glmer’ function in the ‘lme4’ package in R (Bates *et al.* 2015). To obtain the significance of each factor, a type II analysis of variance was conducted as described above, and a *post-hoc* test was conducted as described above.

The visiting frequency of pollinators was analysed using linear mixed-effects models to investigate the effects of pollinator on visiting frequency. Before statistical tests, visiting frequency was transformed with the log_10_ (x + 1) to improve the normality of the data (Zar 2010). Subpopulation, pollinator and their interactions were used as predictive variables, visiting frequency as response variable and different days for observation were included as random factors. The analyses were conducted using the ‘lmer’ function in the ‘lme4’ package (Bates *et al.* 2015). To obtain the significance of subpopulations or pollinator groups, a type II analysis of variance was conducted as described above and a *post-hoc* test was conducted as described above.

All pollen dispositions were analysed using GLMMs with a poisson distribution using the ‘log’ link function. To investigate the effects of stigma morphology, pollinator, and their interaction on pollen deposition on stigma after a single visit, the number of legitimate and illegitimate pollen were modelled. Stigma morphology, pollinators and their interaction were included in the models as fixed factors, and plant nested within different days for observation were defined as random factors. To investigate the effects of clonal fragment size on pollen transfer, the number of pollen (legitimate or illegitimate pollen) was modelled. Sampling location (pollination distance) was used as a fixed factor, and ramet nested within different days was defined as a random factor. To investigate the effects of mating type ratio on pollen transfer, the number of pollen (legitimate or illegitimate pollen) was modelled. Stigma morphology and mating type ratio were specified as fixed factors, and ramets nested within different days for observation were defined as random factors. These analyses were conducted using the ‘glmer’ function in the ‘lme4’ package in R (Bates *et al.* 2015). The overdispersion was checked using the ‘check_overdispersion’ function in the ‘performance’ package (Lüdecke *et al.* 2021). Poisson models included an observation-specific random effect to account for potential overdispersion. The significance of each factor was tested with type II analysis of deviance, and a *post-hoc* test was conducted as described above.

Correlations between stigma-anther separation and pollen grain count or fruit sets for both mating morphs, as well as the relationships between mating type ratios, stigma pollen depositions and fruit sets in clonal patches, were explored through Pearson or Spearman correlation analysis.

All statistical analyses in the present study were performed using the R v4.2.3 (R Core Team 2023), excluding the chi-square test and correlation analysis, which were performed using IBM SPSS Statistics 20 (IBM Corp., Armon, NY, USA). All figures were created using ArcMap 10.8 (ESRI, Redlands, CA, USA) or MS Excel 2010 (Microsoft Corp., Redmond, WA, USA). The data are presented as the mean ± SE.

## Results

### Heterostylous syndrome and clonal growth

#### Floral morph composition and frequency of the population

**Figure 3.**
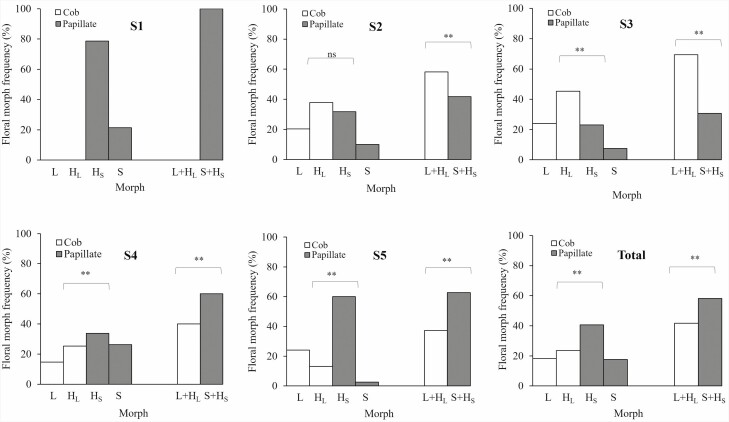
Floral morph composition and frequency of *Limonium otolepis* in different subpopulations. **P < 0.01, *P < 0.05, ns *P* > 0.05.

After examining floral composition and frequency, we found that in the S2–S5 subpopulations, L-morph, S-morph and H-morph (H_L_-morph and H_S_-morph) flowers (Fig. 1C–E) coexisted. However, there were only two distinct pollen/stigma morphology types among these floral morphs. In contrast, the S1 subpopulation had a monomorphic pollen/stigma morphology, comprising H_S_- and S-morph flowers. In the S2 and S3 subpopulations, L- and H_L_-morph flowers dominated, greatly outnumbering the S- and H_S_- morph flowers (χ^2^ = 3.945, *P* = 0.047; χ^2^ = 24.025, *P* < 0.001, respectively). The S4 and S5 subpopulations were dominated by S- and H_S_-morph flowers, with significant differences in the frequency compared with L- and H_L_-morph flowers (χ^2^ = 70.402, *P* < 0.001; χ^2^ = 52.633, *P* < 0.001, respectively) (Fig. 3).

#### Distribution pattern and clonal strength of genet

The population had a patchy distribution due to clonal propagation. Our global positioning system-based investigation revealed significant differences among the five subpopulations in terms of population size, genet distribution, clonal strength, floral morph composition and frequency (Fig. 2). The S1 subpopulation solely consisted of S- and H_S_-morphs with a single stigma/pollen morphology, while the other four subpopulations included L-, S- and H-morphs with dimorphic stigma/pollen morphology. Among these subpopulations, there was a significant difference in the number of genets with the two types of stigma morphologies (χ^2^ = 14.329, *P* < 0.001), and the clonal strength was consistent (χ^2^ = 0.032, *P* = 0.857) in the S4 subpopulation. In the S2 subpopulation, clonal strength significantly differed (χ^2^ = 4.083, *P* = 0.043), but the number of genets with the two types of stigma morphologies did not (χ^2^ = 1.256, *P* = 0.262). The S3 and S5 subpopulations showed no significant differences in clonal strength either the number of genets with the two types of stigma morphology or clonal strength (Table 1).

**Table 1.** Clonal growth of *Limonium otolepis* in different subpopulations.

Subpopulations	No. of genets (Cob, Papillate)	χ^2^	df	*P*-values	Clonal strength (Cob, Papillate)	χ^2^	df	*P*-values
S1	0, 12	-	-	-	0, 19	-	-	-
S2	16, 23	1.256	1	0.262	17, 31	4.083	1	**0.043**
S3	11, 8	0.474	1	0.491	21, 11	3.125	1	0.077
S4	107, 170	14.329	1	<**0.001**	15, 16	0.032	1	0.857
S5	30, 31	0.016	1	0.898	17, 27	2.273	1	0.132

Bold indicates that the significance (*P*-value) is less than 0.05.

#### Ancillary polymorphism and flower size parameter

The pollen and stigma morphologies of *L. otolepis* were dimorphic (Fig. 1I–L,  **[see Supporting Information—Figure S1]**). Pollen grains across all floral morphs were round, with a reticulate surface pattern and three germination holes. The stigma featured a filiform structure, characterized by either cob-like or papillate epidermal cells. In L- and H_L_-morph flowers, both the pollen and stigma shared a coarse-reticulated pollen outer wall and cob-like stigma epidermal cells (Fig. 1I–J). In contrast, S-morph and H_S_-morph flowers exhibited identical fine-reticulated pollen outer walls and papillate stigma epidermal cells (Fig. 1K and L).

In the S4 subpopulation, there were no differences in the total flower length, corolla length, and corolla tube length among the L-, S-, H_L_- and H_S_-morphs. The corolla opening diameter of the S- or H_S_-morph flowers was larger than that of the H_L_-morph flowers (S vs H_L_: *P* = 0.010; H_S_ vs H_L_: *P* = 0.010). The corolla tube diameter of H_S_-morph flowers was larger than that of L- or H_L_-morph flowers (H_S_ vs L: *P* = 0.006; H_S_ vs H_L_: *P* = 0.012). The stigmas and anther heights of the four floral morphs also differed significantly (stigma: Wald χ^2^ = 209.410, df = 3, *P* < 0.001; anther: Wald χ^2^ = 60.452, df = 3, *P* < 0.001). There were significant differences between the high sexual organs (Wald χ^2^ = 8.309, df = 1, *P* = 0.004) and between low sexual organs (Wald χ^2^ = 13.474, df = 1, *P* < 0.001) of the L- and S-morphs, with weak reciprocity. However, stigma-anther separation (L-morph: 0.96 ± 0.03 mm; S-morph: 1.02 ± 0.04 mm) showed no significant difference (Wald χ^2^ = 0.002, df = 1, *P* = 0.9628). The H_L_ and H_S_ morphs had stigma and anther heights between those of the L- and S-morphs (Table 2).

**Table 2.** Floral characteristics parameters of *Limonium otolepis* in the S4 subpopulation (mean ± SE).

Floral traits	L-morph (*n* = 30–45)	H_L_-morph (*n* = 30–45)	H_S_-morph (*n* = 30–45)	S-morph (*n* = 30–45)
Flower length (mm)	4.42 ± 0.09^a^	4.55 ± 0.05^a^	4.55 ± 0.06^a^	4.53 ± 0.06^a^
Corolla length (mm)	4.10 ± 0.04^a^	4.23 ± 0.05^a^	4.21 ± 0.05^a^	4.17 ± 0.06^a^
Corolla tube length (mm)	2.51 ± 0.03^a^	2.59 ± 0.03^a^	2.59 ± 0.03^a^	2.54 ± 0.04^a^
Corolla opening diameter (mm)	2.45 ± 0.06^ab^	2.41 ± 0.06^b^	2.68 ± 0.07^a^	2.70 ± 0.06^a^
Corolla tube diameter (mm)	0.98 ± 0.02^b^	1.00 ± 0.02^b^	1.09 ± 0.02^a^	1.03 ± 0.02^ab^
Stigma height (mm)	4.76 ± 0.06^a^	4.21 ± 0.05^b^	4.02 ± 0.05^b^	3.52 ± 0.04^c^
Anther height (mm)	3.80 ± 0.06^c^	3.92 ± 0.04^c^	4.21 ± 0.04^b^	4.54 ± 0.04^a^
No. of ovules per flower	1	1	1	1

Different superscripted letters indicate significant differences between the morphs (*P* < 0.05).

**Figure 4.**
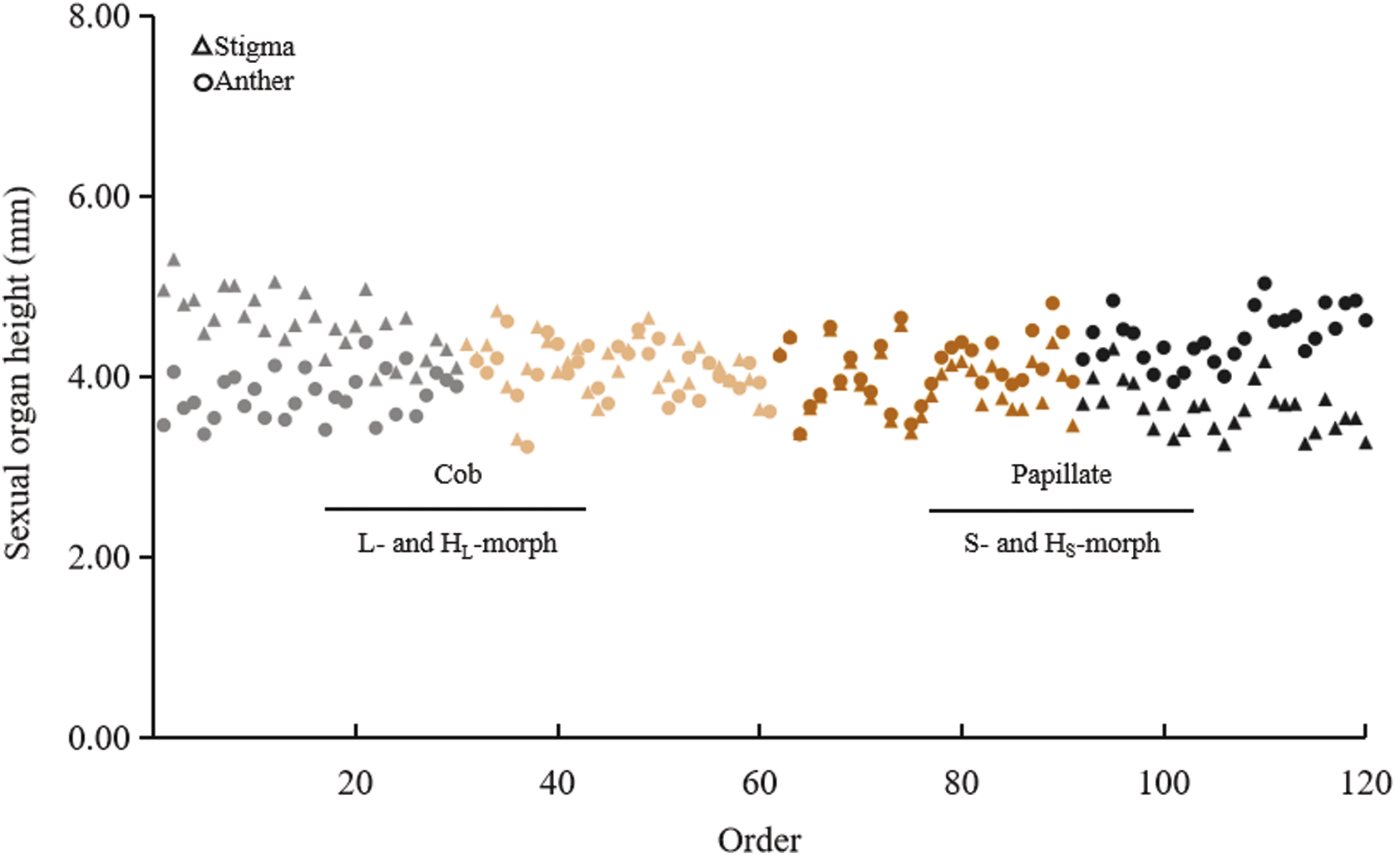
Distribution of sexual organ height of *Limonium otolepis* in the S4 subpopulation.

**Figure 5.**
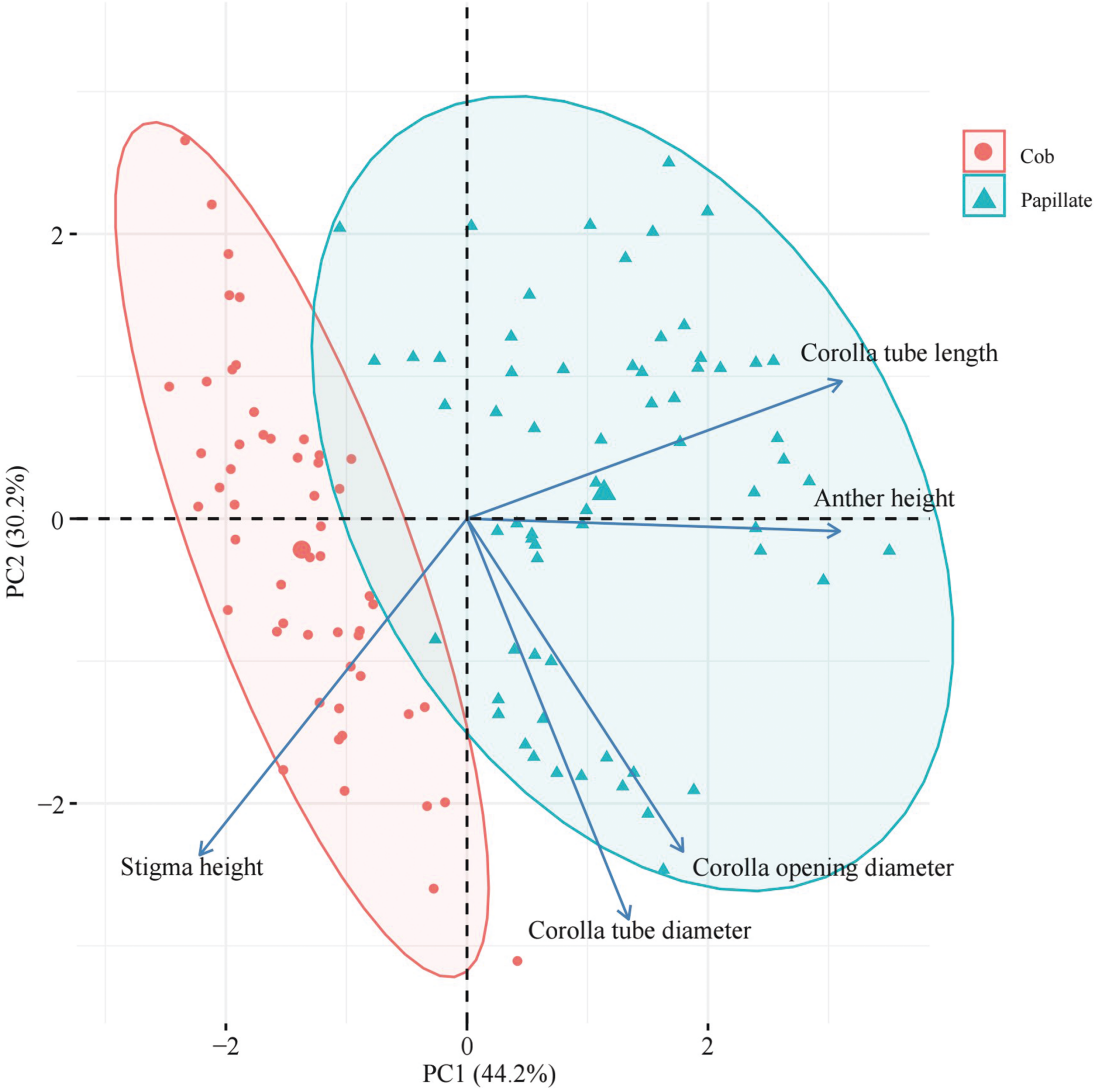
Principal component analysis of five floral traits in *Limonium otolepis.*

Random measurements of stigma and anther heights from 30 ramets, encompassing 120 flowers within the mixed clonal patch, revealed some consistency in stigma and anther distribution between the two stigma morphology types. L- and S-morph flowers constituted a smaller proportion, while transitional stigma and anther heights, along with those of the H-morph, were more prevalent (Fig. 4). PCA of the five floral characteristics resulted in two clusters corresponding to the two stigma morphology types (Fig. 5). PC1 showed a significant distinction between them, primarily driven by anther height, corolla tube length and stigma height. Anther height was the most influential, exhibiting a negative correlation with stigma height. Regarding clustering, flowers with papillate stigma morphology displayed more pronounced differences in anther height, corolla tube length and stigma height than those with cob-like stigmas.

#### Heteromorphic incompatible systems

**Figure 6.**
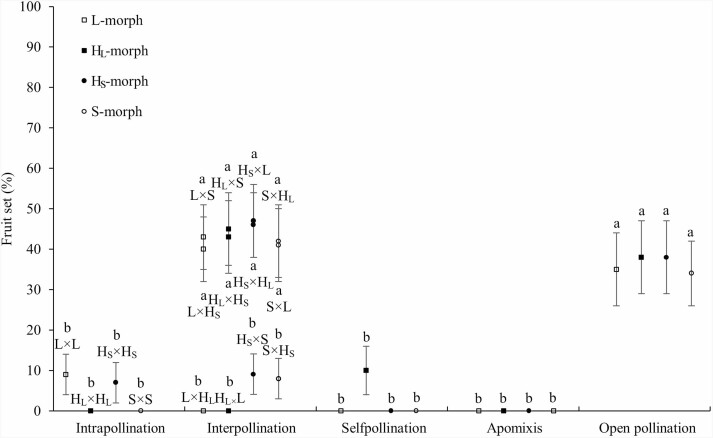
Fruit set of floral morphs under different treatments of pollination in *Limonium otolepis.* The different lowercase letters indicate significant differences between the treatments or morphs (*P* < 0.05).

*Limonium otolepis* exhibited no apomixis, self- or intramorphic compatibility. ‘Intermorph pollination’ was compatible between floral morphs with different pollen and stigma morphologies, such as L × S, L × H_S_, H_L_ × S and H_L_ × H_S_, and vice versa, such as Hs × S, S × H_S_, L × H_L_, H_L_ × L (Fig. 6). In all pollination trearment, the fruit set did not depend on the parent stigma morphology and the interaction of parent stigma morphology and treatment (Wald χ^2^ = 0.012, df = 1, *P* = 0.914; Wald χ^2^ = 3.808, df = 6, *P* = 0.723), but on the treatment (Wald χ^2^ = 111.737, df = 6, *P* < 0.001). Under open pollination, the fruit sets of L-, H_L_-, H_S_- and S-morphs were 35.48 ± 8.59 %, 37.50 ± 8.56 %, 37.50 ± 8.56 %, and 34.38 ± 8.40 %, respectively, with no significant difference (Wald χ^2^ = 0.089, df = 3, *P* = 0.993) (Fig. 6).

A controlled pollination experiment in the S1 subpopulation demonstrated strict self-incompatibility. The fruit sets for intramorph pollination, artificial self-pollination and apomixis were 3.92 ± 2.75 %, 0 %, and 0 %, respectively. These three treatments showed no significant differences (Wald χ^2^ = 1.714, df = 2, *P* = 0.424). Open pollination was 12.20 ± 2.56 %, which was comparable to intramorph pollination (*P* = 0.310), artificial self-pollination (*P* = 0.300) or apomixis (*P* = 0.338).

### Legitimate pollen transfer mediated by pollinator functional group

#### Pollinators and visiting frequency

**Figure 7.**
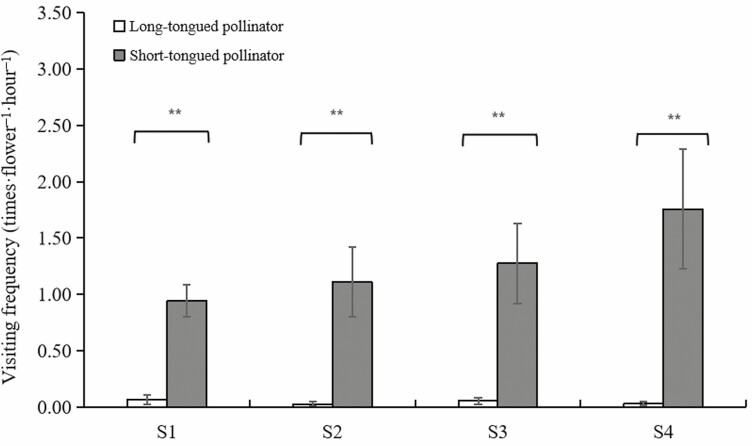
Visiting frequency of long-tongued and short-tongued insects in different subpopulations of *Limonium otolepis.* **P < 0.01, *P < 0.05, ns *P* > 0.05.

We have carried out 6–26 h and 13–55 times of observation in S1–S4 subpopulations, respectively. A total of seven genera of insects were observed, which ST insects were the main pollinator groups, such as *Syrphidae* sp., *Muscidae* sp. and *Halictus* sp. (Fig. 1F–H). The visiting frequencies of the ST insects in the S1–S4 subpopulations were 0.942 ± 0.141, 1.110 ± 0.310, 1.275 ± 0.356, and 1.759 ± 0.531 times flower^−1^h^−1^, respectively (Wald χ^2^ = 2.358, df = 3, *P* = 0.501), and that of LT insects were 0.066 ± 0.039, 0.027 ± 0.023, 0.055 ± 0.03, and 0.031 ± 0.015 times flower^−1^h^−1^, respectively (Wald χ^2^ = 1.571, df = 3, *P* = 0.666). There were significant differences in each subpopulation between LT and ST insects (S1: *P = *0.004; S2: *P* < 0.001; S3: *P* < 0.001; S4: *P* < 0.001) (Fig. 7).

#### Pollen deposition after single visit

**Figure 8.**
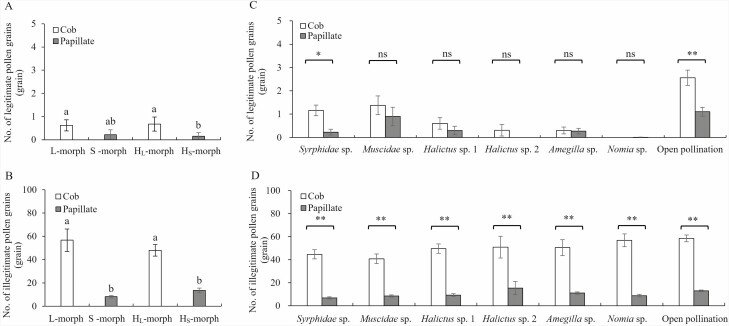
Pollen transfer of *Limonium otolepis* after a single visit by different pollinators in the S4 subpopulation. (A), (B) Pollen transfer of different floral morphs after a single visit of short-tongued pollinators; (C), (D) Pollen transfer of two mating types after a single visit of different short-tongued pollinators. Lowercase letters indicate a significant difference between the morphs (*P* < 0.05). **P < 0.01, *P < 0.05, ns *P* > 0.05.

As ST pollinators were the primary visitors in each subpopulation, we investigated pollen deposition on stigmas of ramets with different floral morphs following single visits by these pollinators. The results highlighted that grain numbers of both of legitimate and illegitimate pollen on stigmas of L- and H_L_-morph were significantly higher than those of S- and H_S_-morphs (see Fig. 8 A and B). To further clarify these distinctions, we assessed the pollen grain count on stigmas carried by the main pollinators, namely *Syrphidae* sp., *Muscidae* sp., *Halictus* sp., *Amegilla* sp. and *Nomia* sp. Our analysis revealed that only *Syrphidae* sp. deposited more legitimate pollen grains on flowers with cob-like stigma morphology than on those with papillate stigma morphology (*P* = 0.004). Conversely, the number of legitimate pollen grains from other pollinators remained consistent between the two mating types (Fig. 8C). However, the number of illegitimate pollen grains was significantly higher in flowers with cob-like stigmas than in those with papillate stigmas (Fig. 8D). This phenomenon likely contributes to the difference in stigmatic pollen deposition between the two mating types during open pollination, where both legitimate and illegitimate stigmatic pollen depositions of mating morphs with cob-like stigma were notably greater than those of mating morphs with papillate stigma (legitimate: Wald χ^2^ = 8.167, df = 1, *P* = 0.004; illegitimate: Wald χ^2^ = 576.393, df = 1, *P* < 0.001).

#### Degree of herkogamy and pollen deposition

The correlation analysis between stigma-anther separation and stigmatic pollen deposition in *L. otolepis* revealed significant negative correlations for floral morphs with cob-like stigmas. These negative correlations were observed between the number of illegitimate pollen grains and absolute stigma-anther separation (ASAS) (*r* = −0.522, *P* < 0.001) or with relative stigma-anther separation (RSAR) (*r* = −0.535, *P* < 0.001), as well as between the number of legitimate pollen grains and ASAS (*r* = −0.245, *P* = 0.010) or RSAS (*r* = −0.218, *P* = 0.023). However, floral morphs with papillate stigma morphology did not exhibit significant correlations between the number of illegitimate pollen grains and ASAS (*r* = −0.195, *P* = 0.053) or RSAS (*r* = −0.136, *P* = 0.180), nor between the number of legitimate pollen grains and ASAS (*r* = −0.080, *P* = 0.432) or RSAS (*r* = −0.065, *P* = 0.523) (Table 3).

**Table 3.** Correlation analyses between stigma–anther separation and pollen deposition of *Limonium otolepis.*

Pollen types	Absolute stigma-anther separation	Relative stigma-anther separation
No. of illegitimate pollen grains on cob-like stigma	−0.522**	−0.535**
No. of legitimate pollen grains on cob-like stigma	−0.245*	−0.218*
No. of illegitimate pollen grains on papillate stigma	−0.195	−0.136
No. of legitimate pollen grains on papillate stigma	−0.080	−0.065

Bold indicates that the significance (*P*-value) of both is less than 0.05. **P < 0.01; *P < 0.05.

### Legitimate pollen transfer mediated clonal growth

#### Impacts of the distance to the nearest compatible mating partner on pollen transfer

**Figure 9.**
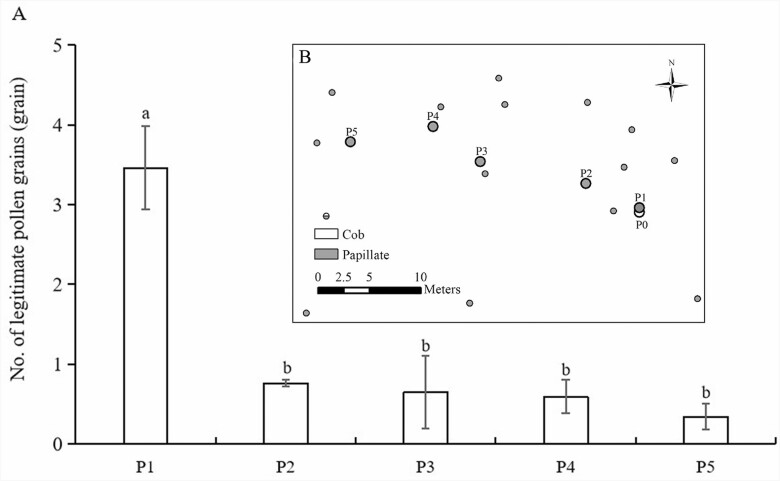
Legitimate pollen dilution of clonal genets in monomorphic patch. (A) The legitimate pollen deposition under different pollination distance to the nearest compatible mating partner in *Limonium otolepis*. Lowercase letters indicate a significant difference between the locations (*P* < 0.05). (B) The block diagram shows genet distribution and sampling location (P1–P5 is the location of the sampling papillate genets, and P0 is the location of the nearest compatible mating partner genets).

In the large independent monomorphic patch composed of flowering ramets with papillate stigma morphology, the number of legitimate pollen on the stigma gradually decreased from the outside to the inside (Fig. 9). The number of legitimate pollen grains on the stigma of P1 closest to the compatible mating partner (P0) was the highest (3.55 ± 0.65 grains). The number of legitimate pollen grains on stigmas of P2–P5 ramets was all significantly lower than that of P1 ramets. The results indicated that the insect pollination distance was short (<10 m), and the size of the genets played an important role in pollen transfer.

#### Impacts of mating type ratio on pollen transfer and sexual reproduction

Pollen transfer and fruit set correlated with floral morph frequencies (see Table 4). For flowers with cob-like stigmas, both legitimate pollen deposition and fruit set were negatively correlated with the mating type ratio. For flowers with papillate stigma, both stigmatic legitimate pollen proportion and fruit set negatively correlated with the mating type ratio (Table 5). When mating type ratios were similar, fruit sets tended to be equal. Deviations in the ratio resulted in a higher fruit set for the mating type with a lower percentage. The comparative analysis of pollen number and fruit set of the two mating types in mixed patches also suggested that the number of illegitimate pollen grains only depended on the stigma morphology of the patch (Wald χ^2^ = 132.887, df = 1, *P* < 0.001). Legitimate pollen grains depended on the proportion of floral morph, patch stigma morphology (Wald χ^2^ = 14.988, df = 1, *P* < 0.001; Wald χ^2^ = 12.981, df = 1, *P* < 0.001). And fruit set only depended on the proportion of floral morph (Wald χ^2^ = 65.414, df = 1, *P* < 0.001).

**Table 4.** Effect of mating type ratio of *Limonium otolepis* on pollen transfer and sexual reproduction.

Patches	Stigma morphology	Mating type ratio (No. ramets)	No. of illegitimate pollen grains	No. of legitimate pollen grains	Fruit set
S1	Papillate	1 (130)	14.63 ± 0.70	0.11 ± 0.02	0.12 ± 0.02
M1	Papillate	0.56 (22)	11.23 ± 1.22	1.41 ± 0.67	0.34 ± 0.03
	Cob	0.44 (17)	43.90 ± 7.28	1.87 ± 0.75	0.38 ± 0.04
M2	Papillate	0.09 (9)	18.50 ± 3.78	0.67 ± 0.33	0.47 ± 0.08
	Cob	0.91 (95)	56.80 ± 3.80	0.28 ± 0.12	0.14 ± 0.02
M3	Papillate	0.59 (24)	27.33 ± 5.23	0.40 ± 0.18	0.39 ± 0.05
	Cob	0.41 (17)	69.28 ± 4.26	1.67 ± 0.42	0.49 ± 0.05
M4	Papillate	0.15 (8)	14.71 ± 2.34	0.79 ± 0.31	0.67 ± 0.21
	Cob	0.85 (47)	39.86 ± 2.55	0.78 ± 0.15	0.23 ± 0.03
M5	Papillate	0.67 (46)	18.26 ± 1.88	0.23 ± 0.07	0.31 ± 0.03
	Cob	0.33 (23)	46.19 ± 4.21	1.52 ± 0.85	0.44 ± 0.05

**Table 5.** Correlation analysis between mating type ratio and pollen transfer or fruit set of *Limonium otolepis.*

Mating type ratio	No. of illegitimate pollen grains	No. of legitimate pollen grains	Proportion of legitimate pollen grains	Fruit set
Ratio of ramets with cob-like stigma	−0.154	−0.920*	−0.383	−0.953*
Ratio of ramets with papillate stigma	−0.037	−0.598	−0.968**	−0894*

Bold indicates that the significance (*P*-value) of both is less than 0.05. **P < 0.01; *P < 0.05.

## Discussion

Heterostyly promotes precise pollen transfer through reciprocal herkogamy between floral morphs (Darwin 1877; Lloyd and Webb 1992; Barrett 2002). Clonal growth creates patchy populations, affecting the spatial distribution of mating types, floral frequency and pollen dispersal, particularly in larger clonal patches (Wang *et al.* 2005; Brys *et al.* 2007). Consequently, heterostyly with clonal growth challenges stable floral composition and frequency (Thompson *et al.* 1998; Wang *et al.* 2005; Brys *et al.* 2007; da Cunha *et al.* 2014). In a study of *Limonium otolepis*, vigorous clonal growth led to patchy population distribution, favouring individuals with papillate stigma morphology. Smaller flowers with shorter corolla tubes attracted ST pollinators like flies and small bees but resulted in uneven pollen transfer between high and low sexual organs, potentially driving H-morph evolution. Clonal growth reduced intermorphic pollen transfer and reproductive fitness, hastening reciprocal herkogamy variation. However, the emergence of the H-morph did not cause the transfer of self or intramorphic incompatibility, indicating compatibility regardless of reciprocal herkogamy. In view of the S1 monomorphic subpopulation’s heteromorphic style syndrome, clonal growth’s reproductive assurance may maintain the heteromorphic incompatibility system.

### Clonal growth and sexual reproduction

Sexual reproduction is essential for angiosperm diversity, enhancing genetic diversity by uniting male and female gametophytes (Richards 1997). Efficient pollen transfer and seedling renewal support sexual reproduction (Alves-dos-Santos 2002; Kéry *et al.* 2003). However, conflicts between clonal growth and sexual reproduction can harm clonal plant fitness, especially in disassortative mating populations (Kéry *et al.* 2003; Brys *et al.* 2007; Barrett 2015).

In the saline desert environment of *L. otolepis*, we observed a distinct patchy distribution of population composed of five subpopulations with varying genet ratios, clonal strength, floral morph compositions and frequencies (Fig. 2, Table 1). For instance, the S1 subpopulation featured only H_S_- and S-morphs with papillate stigma morphology, while the S2–S5 subpopulations were dimorphic, comprising L-, S- and H-morphs with two stigma morphologies, exhibiting significant frequency differences. Some subpopulations had uniform genet ratios but differing clonal strength, while others displayed uneven genet ratios with uniform clonal strength. Such disparities may originate from the ‘founder effect’ during fragmented population formation due to human activities, and differing clonal strength may be linked to the prevalence of sexual or asexual reproduction. This phenomenon reflects the complex interplay between the clonal growth and sexual reproduction, especially the effect of sexual reproduction on clonal strength. A survey involving 15 young individuals indicated no seedling replenishment, possibly due to the salinized desert environment, which may foster clonal growth. Thus, the influence of sexual reproduction on clonal growth is multifaceted, with vigorous clonal growth reciprocally affecting sexual reproduction.

We studied the impact of clonal fragments on pollen transfer within a larger clonal patch of the same mating types. We observed that the proximity of ramets to compatible mating partners outside the patch resulted in significantly higher legitimate pollen deposition on stigmas compared to ramets that were more centrally located (Fig. 9). This finding indicates that clonal patches dilute legitimate pollen flow. Our investigation also encompassed pollinators and their visiting behaviour. We discovered that most pollinators in these populations were ST insects such as flies and small bees. These insects primarily transferred pollen between nearby or even the same ramets, with a few exceptions, such as LT insects. Shorter pollination distances exacerbated the impact of clonal patches on legitimate pollen transfer. In our raw data on stigmatic pollen deposition following a single visit, we noted that instances where stigmatic pollen deposition was absent accounted for 59–100 % of the data. These findings highlight the combined influence of clonal growth and pollinator behaviour on pollen transfer and sexual reproduction. Effective, legitimate pollen transfer is closely linked to pollinator visiting behaviour (Eckert 2000; Wang *et al.* 2005). Similar phenomena have been observed in some heterostylous plants with clonal growth. For instance, in tristylous *Lythrum salicaria*, ‘long-distance’ bee-mediated pollen flow maintained intermorph mating (Balogh and Barrett 2018).

To investigate the impact of clonal growth-induced uneven floral morph ratios on pollen transfer and sexual reproduction, we performed a correlation analysis between floral morph frequencies and legitimate pollen deposition within various mixed clonal patches. The findings revealed that patches with relatively balanced mating type ratios or those with the mating type with a lower representation within a patch exhibited higher fruit set rates. However, in the monomorphic S1 subpopulation (the largest monomorphic clonal patch), which featured only one mating type, the number of legitimate pollen grains on stigmas (0.11 ± 0.02 grains) and the resulting fruit set (0.12 ± 0.02) were notably lower than all other investigated clonal patches. This phenomenon aligns with findings from studies on clonal, distylous aquatic plants such as *Hottonia palustris* and *N. peltata* (Wang *et al.* 2005; Brys *et al.* 2007), both of which illustrate the impact of clonal patches with uneven floral morph frequencies on pollen transfer and sexual reproduction. These studies also highlight the role of negative frequency-dependent selection in mitigating disparities in floral morph frequencies. In summary, there exists a multifaceted interplay between clonal growth and sexual reproduction in *L. otolepis*. Clonal growth exacerbates deviations in mating type frequencies, resulting in distinct patchy distributions in subpopulations, unequal pollen flow, and varying sexual reproduction fertility within and outside the patches. In contrast, clonal growth is reciprocally influenced by sexual reproduction, including limited seedling renewal and the presence of negative frequency-dependent selection.

### Floral morph variation and pollen transfer

While heterostyly is common across 28 angiosperm families, most species share characteristic floral traits, such as medium-sized flowers, a radiate corolla and an extended corolla tube (Ganders 1979). Due to the corolla tube’s selectivity toward pollinators (Arroyo and Dafni 1995; Arroyo *et al.* 2002; Simón-Porcar *et al.* 2014), ST insects tend to be ineffective pollinators, frequently leading to asymmetric pollen transfer between mating types or variations in floral morphs (Thompson *et al.* 1998; Alves-dos-Santos 2002; Pérez-Barrales and Arroyo 2010; Santos-Gally *et al.* 2015; Zhu *et al.* 2015; Raupp *et al.* 2020; Furtado *et al.* 2021). In the case of *L. otolepis,* its smaller flowers with shorter corolla tubes lower the threshold for ST insects, making flies and small bees more efficient pollinators (visiting frequency: 0.942–1.759 flower·visits^-1^h^-1^) (Fig. 1F–H), compared to LT pollinators such as butterflies and beeflies (visiting frequency: 0.027–0.066 flower·visits^−1^h^−1^). Nonetheless, the asymmetrical pollen flow between high–low sexual organs still leads to floral morph variation.

Within a patch featuring an equal mating-type frequency, we examined pollen deposition on stigmas of the two mating types after single visits by ST insects. The results indicated a significantly greater legitimate and illegitimate pollen deposition count on stigmas with cob-like stigma morphology than on those with papillate stigma morphology. Subsequent investigation identified the *Syrphidae* sp. as the sole insect delivering more legitimate pollen to flowers with cob-like stigma than those with papillate stigma. Meanwhile, a correlation analysis revealed a significant negative correlation between stigma-anther separation and legitimate pollen count on cob-like stigma (see Table 3), in which H-morphs with minimal stigma-anther separation received more legitimate pollen. However, the floral morphs with papillate stigma displayed no significant correlation, while H-morphs with papillate stigma morphology also received more legitimate pollen than S-morphs. The mainly low legitimate pollen count on papillate stigma (accounting for 38 % of zero pollen depositions) possibly influenced the correlation analysis results. Based on these findings, H-morphs enjoy an advantage in pollen transfer due to their higher legitimate pollen count on stigma, irrespective of whether the floral morph features cob-like or papillate stigmas.

Due to the need for reproductive assurance, classical homostyly varies in phenotype exhibiting smaller flowers with a capacity for autonomous self-pollination (Zhou *et al.* 2015, 2017; Barrett 2019). However, we do not see the selective forces promoting self-pollination in *L. otolepis*. At first, there are no higher stigma illegitimate pollen (self-pollen and intramorph pollen) counts in H_L_-morph or H_S_-morph flowers than in L-morph flowers or S-morph flowers (Fig. 8B). Secondly, the number of illegitimate pollen grains was significantly higher in H-morph with cob-like stigmas (H_L_) than in those with papillate stigmas (H_S_) following single visits by pollinators (Fig. 8B). These results indicate that the illegitimate stigmatic pollen depositions of the H-morph flower were not only affected by stigma-anther separation but also the heteromorphism of pollen-stigma morphology. This phenomenon has also been supported by relevant literature and views (Dulberger 1992; Costa *et al.* 2017).

### The formation and adaptability of H-morph

The classical homostyly has attracted considerable attention in the evolution of heterostyly (Jiang *et al.* 2018; Wang *et al.* 2020; Charlesworth 2023; Mora-Carrera *et al.* 2023). It occurs frequently and is often accompanied by the breakdown of incompatible heteromorphic systems (Ganders 1979; Barrett 1992; Barrett and Shore 2008; Cohen 2010; Huu *et al.* 2016, 2022; Hoshino *et al.* 2022). In the *L. otolepis* population, based on the floral traits and the self-incompatibility relationship between floral morphs, H-morph flowers differed from the classical homostyly reported. Compared with L- and S-morph flowers, H-morph flowers overcame the disadvantage of low sexual organ retraction and improved the ability to accept legitimate S-morph flowers when pollinated by *Syrphidae* sp. or dispersed pollen for L-morph flowers.

Distylous *L. otolepis*, characterized by its short corolla tube and small flowers, exhibits an asymmetric disassortative pollen transfer pattern driven by ST pollinators and clonal growth. However, the selective pressure for H-morph formation may be reduced due to lower ovule counts, increased ST pollinator visits, and robust clonal growth. Notably, the impact of clonal growth on floral variation may work in both directions. It could encourage H-morph formation by reducing pollen transfer efficiency within clonal patches and might diminish the selective pressure for H-morph formation by alleviating the maintenance burden of sexual reproduction. Considering these factors, the variation in floral morphs or the emergence of H-morphs may align with an evolutionary model of ‘relaxed selection’ (Lahti *et al.* 2009; Day and Kokko 2015), potentially influenced by unlinked modifier genes.

Reciprocal herkogamy, ancillary pollen/ stigma polymorphism, and heteromorphic incompatibility constitute a heterostylous syndrome (Ganders 1979; Dulberger 1992). Reciprocal herkogamy is pivotal in ensuring precise, legitimate pollen transfer and preserving the heteromorphic incompatibility system (Zhou *et al.* 2015). Changes or losses in reciprocal herkogamy often coincide with alterations in heteromorphic incompatibility in various plant species (Yuan *et al.* 2017; Zhong *et al.* 2019; Huu *et al.* 2022). For instance, Huu *et al.* (2022) identified that the cytochrome enzyme P450 *CYP734A50* gene controls style length and female self-incompatibility in *Primula* plants. Notably, in *L. otolepis*, variations in reciprocal herkogamy between floral morphs did not lead to alterations in heteromorphic incompatibility, even in the monomorphic S1 subpopulation where H-morphs predominated. Floral morphs with varying pollen ornamentation and stigma papilla cell morphologies remained compatible, regardless of reciprocal herkogamy. Variations in reciprocal herkogamy and shifts in physiological incompatibility may have evolved independently. This phenomenon has also been reported by phylogenetic reconstructions based on 121 species in Plumbaginaceae (Costa *et al.* 2019).

In conclusion, this study explored the roles of pollinators and clonal growth in pollen transfer, H-morph occurrence, and related influencing factors in a distylous plant with a clonal growth habit, small flowers, and a fragmented population. Given the diversity of pollination systems and the complex relationship between sexual and clonal reproduction, further research is warranted. Topics such as understanding reciprocal herkogamy’s function in small flowers, investigating its link to heteromorphic incompatibility, and unravelling H-morph formation will contribute to a more comprehensive understanding of these intricate interactions.

## Supporting Information

The following additional information is available in the online version of this article –

**Figure S1.** Stigma-pollen morphology of *Limonium otolepis*. (A) pollen grains and stigma morphology of L-morph (with cob-like stigma epidermal cells and coarse-reticulated pollen outer wall); (B) pollen grains and stigma morphology of S-morph (with papillate stigma epidermal cells and fine-reticulated pollen outer wall); (C) pollen grains and stigma morphology of H_L_-morph (with cob-like stigma epidermal cells and coarse-reticulated pollen outer wall); (D) pollen grains and stigma morphology of H_S_-morph (with papillate stigma epidermal cells and fine-reticulated pollen outer wall).

plae020_suppl_Supplementary_Data_S1

plae020_suppl_Supplementary_Data_S2

plae020_suppl_Supplementary_Data_S3

plae020_suppl_Supplementary_Data_S4

plae020_suppl_Supplementary_Data_S5

plae020_suppl_Supplementary_Data_S6

plae020_suppl_Supplementary_Data_S7

plae020_suppl_Supplementary_Data_S8

plae020_suppl_Supplementary_Figure_S1

## Data Availability

The data underlying this article are available as Supporting Information.
