# Supplementary material for: Floral morph variation mediated by clonal growth and pollinator functional groups of Limonium otolepis in a heterostylous fragmented population
Source: AoB Plants. 2024 Mar 27;16(2):plae020. doi: 10.1093/aobpla/plae020 (PMC11041057; doi:10.1093/aobpla/plae020)
Supplement: plae020_suppl_Supplementary_Figure_S1 [file plae020_suppl_supplementary_figure_s1.pdf]

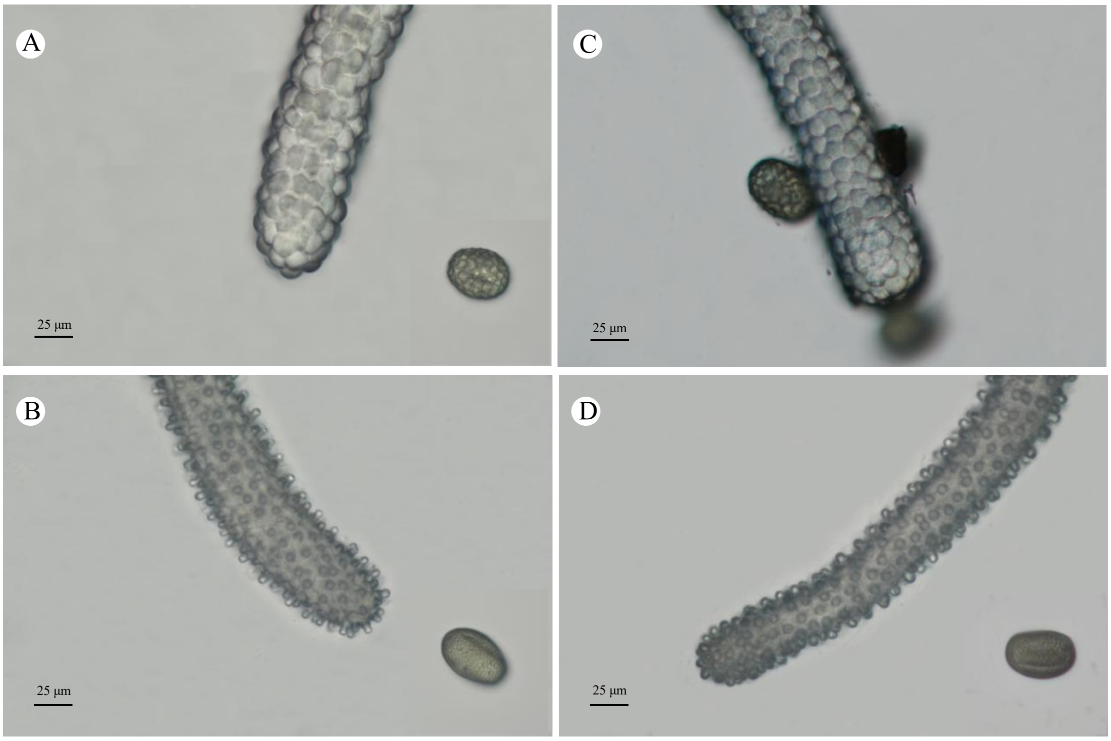

Figure S1. Stigma-pollen morphology of *Limonium otolepis*. (A) pollen grains and stigma morphology of L-morph (with cob-like stigma epidermal cells and coarse-reticulated pollen outer wall); (B) pollen grains and stigma morphology of S-morph (with papillate stigma epidermal cells and fine-reticulated pollen outer wall); (C) pollen grains and stigma morphology of H_L-morph (with cob-like stigma epidermal cells and coarse-reticulated pollen outer wall); (D) pollen grains and stigma morphology of H_S-morph (with papillate stigma epidermal cells and fine-reticulated pollen outer wall).
